# The Asymmetry Observed between the Effects of Photon–Phonon Coupling and Crystal Field on the Fine Structure of Fluorescence and Spontaneous Four-Wave Mixing in Ion-Doped Microcrystals

**DOI:** 10.3390/nano14080671

**Published:** 2024-04-12

**Authors:** Huanrong Fan, Zhongtai Zhang, Iqbal Hussain, Qinyue Yang, Muhammad Kashif Majeed, Muhammad Imran, Faizan Raza, Peng Li, Yanpeng Zhang

**Affiliations:** 1 Key Laboratory for Physical Electronics and Devices of the Ministry of Education & Shaanxi Key Lab of Information Photonic Technique, Xi’an Jiaotong University, Xi’an 710049, China; fanhuanrong@hut.edu.cn (H.F.); iqbal479@gmail.com (I.H.); 2023022346@m.scnu.edu.cn (Q.Y.); mkashif@stu.xjtu.edu.cn (M.K.M.); imran89@mail.xjtu.edu.cn (M.I.); faizanraza17@outlook.com (F.R.); 2 College of Electrical and Information Engineering, Hunan University of Technology, Zhuzhou 412007, China; 3 School of Resource & Environment and Safety Engineering, Hunan University of Science and Technology, Xiangtan 411201, China; zzhongtai0301@163.com; 4 State Key Lab of Modern Optical Instrumentation, Centre for Optical and Electromagnetic Research, College of Optical Science and Engineering, International Research Center for Advanced Photonics, Zhejiang University, Hangzhou 310058, China; 5 Center for Regenerative and Reconstructive Medicine, Med-X Institute, The First Affiliated Hospital of Xi’an Jiaotong University, Xi’an 710061, China

**Keywords:** asymmetry, photon–phonon dressing, crystal field splitting

## Abstract

In this paper, we explore the asymmetry observed between the effects of photon–phonon coupling (nested-dressing) and a crystal field (CF) on the fine structure of fluorescence (FL) and spontaneous four-wave mixing (SFWM) in Eu^3+^: BiPO_4_ and Eu^3+^: NaYF_4_. The competition between the CF and the strong photon–phonon dressing leads to dynamic splitting in two directions. The CF leads to static splitting in one direction under weak phonon dressing. The evolution from strong dressing to weak dressing results in spectral asymmetry. This spectral asymmetry includes out-of-phase FL and in-phase SFWM. Further, the large ratio between the dressing Rabi frequency and the de-phase rate leads to strong FL and SFWM asymmetry due to photon–phonon constructive dressing. Moreover, the experimental results suggest the analogy of a spectra asymmetry router with a channel equalization ratio of 96.6%.

## 1. Introduction

In recent years, researchers have made remarkable advances in regulating quantum coherence excitation in atomic mediums. This process has led to many potentially important applications, including all-optical routers [1,2], quantum memory [3,4], fluorescence resonance imaging [5,6,7,8,9,10] and the tracking of single upconversion nanoparticles [11]. Bismuth phosphate (BiPO_4_) has drawn significant attention as a host medium for doping lanthanide ions [12,13,14] in luminescent applications. In general, the luminescent properties of phosphors are highly influenced by the crystal structure of the host material. Europium ions are considered to be strong spectroscopic probes in various host materials because of their multiplet structure with nondegenerate first-excited (^5^D_0_) and ground (^7^F_1_) levels, respectively. Moreover, the intensity ratio of the ^5^D_0_→^7^F_J_ transition and its Stark components are helpful for understanding the local symmetry [15] at the Eu^3+^ site. Temperature always plays a vital role. Many studies have revealed a strong coupling of phonons with the magnetic degree of freedom reflected in the renormalization of the phonon self-energy parameters. The low-frequency phonons become sharper, while high-frequency phonons show a broadening attributed to the additional available magnetic damping channels [16].

The symmetry of out-of-phase FL and in-phase SFWM has been explored in a variety of atomic media [17,18]. The dressing effect can be manipulated via detuning of the frequency and attenuations of the laser power to control the lifetime of FL and SFWM processes in atomic-like media. The asymmetry of the crystal field (CF) with out-of-phase FL has also been explored [19,20]. However, previous reports on this asymmetry have only considered dressing or CF.

In this study, we investigated the asymmetry of dressing and CF splitting with out-of-phase fluorescence and in-phase SFWM by changing the time gate width and time gate position in nanocrystals, taking Eu^3+^: BiPO_4_ and Eu^3+^: NaYF_4_ as examples. Further, the photon–phonon dressing dynamic splitting makes ^7^F_1_ split into three levels in two directions. However, the CF static splitting leads ^7^F_1_ to split into two energy levels in one direction (x or z). Moreover, the competition between the photon–phonon dressing and CF splitting leads to spectral asymmetry, whereas asymmetry evolves from out-of-phase FL to in-phase SFWM. Finally, we showed that FL asymmetry is sensitive to the time gate width while SFWM asymmetry is sensitive to the time gate position. The large dressing Rabi frequency and small de-phase rate result in strong FL and SFWM asymmetry.

## 2. Experimental Scheme

In this experiment, the Eu^3+^-doped BiPO_4_ sample has a molar ratio of 12:1, obtained by utilizing a combination of the pure hexagonal phase (HP) and a fixed concentration of the low-temperature monoclinic (LTMP) of 5% Eu^3+^ in each sample. The HP corresponds to C_2_ symmetry, and LTMP corresponds to C_1_ symmetry. A larger hexagonal phase refers to more high-frequency phonons due to its low crystal symmetry. The phonons increase due to the enhancement in the concentration of the hexagonal phase [21]. The mixed phases (more HP and less LTMP) of (near 0.5:1) Eu^3+^: BiPO_4_ relates to the C_2_ symmetry. The (1:1/4) Eu^3+^: NaYF_4_ corresponds to the Cs symmetry.

**Figure 1:**
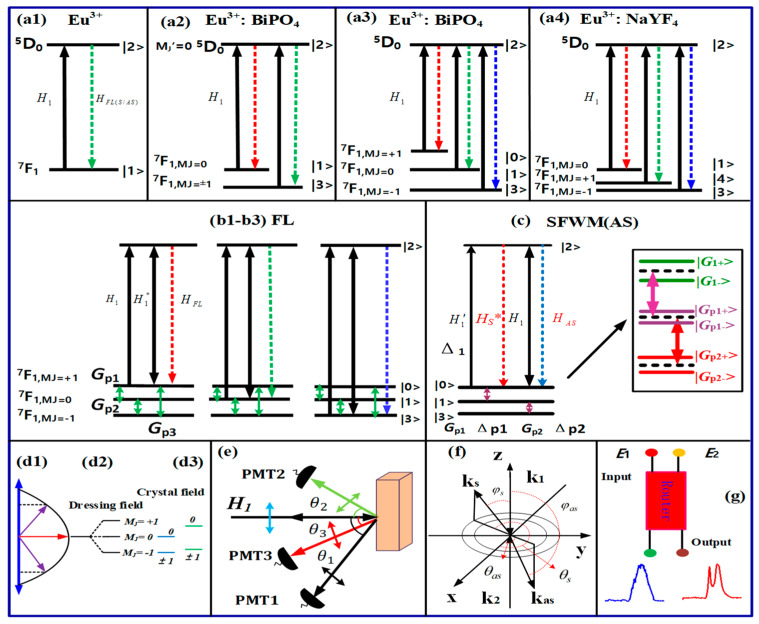
(**a1**) Original energy level in the free ion state Eu^3+^. (**a2**) Crystal field splitting of the ^7^F_1_ energy level for Eu^3+^: BiPO_4_ (pure crystal splitting). (**a3**) Dressing Zeeman-like splitting (|−1>, |0>, |1>) in Eu^3+^: BiPO_4_ (dressing-assisted crystal field splitting). (**a4**) Crystal splitting in Eu^3+^: NaYF_4_. (**b**) The FL signal generation process in a three-level system. (**b1**) The transitions between ^5^D_0_ and ^7^F_1, mj = +1_; (**b2**) the transitions between ^5^D_0_ and ^7^F_1, mj = 0_; (**b3**) the transitions between ^5^D_0_ and ^7^F_1, mj = −1_. (**c**) The SFWM signal generation process in a three-level system. (**d1**) Directional precession splitting, (**d2**) dressing field and (**d3**) crystal field. (**e**) Schematic diagram of the experimental setup with polarization angle. (**f**) SFWM phase matching schematic with different angles. (**g**) Asymmetry spectra router model.

The scheme of the experiment is shown in Figure 1e. The sample temperature was 300 K. We deployed a tunable dye laser (line width of 0.04 cm^−1^) driven by an injection-locked single-mode Nd: YAG laser (Continuum Power lite DLS 9010, 10 Hz repetition rate, 5 ns pulse width) to produce the pumping field *H*_1_ (ω_1_, Δ_1_). The pumping field *H*_1_ excited the sample and was reflected back from the crystal surface in its original path, which is named ${E^{\prime }}_{1}$, with a small angle between them.

The transition of energy levels from the ground-state ^7^F_1_ to excited-state ^5^D_0_ is shown in Figure 1(a1). The fine structure of the Eu^3+^-doped BiPO_4_ crystal was used for the ^7^F_1_→^5^D_0_ transition. The two states, the ground state ^7^F_1_ and the excited state ^5^D_0_, are shown in Figure 1(a2). The ground state ^7^F_1_ is split into M_J_ = 0 and M_J_ = ±1. Figure 1(a3) shows the dressing of Zeeman-like splitting in Eu^3+^: BiPO_4_. Figure 1(a4) shows the crystal splitting of Eu^3+^: NaYF_4_. Figure 1(b1–b3) outlines the effect of interaction between photons and phonons on the initial energy levels of ^7^F_1_, as it is observed in the fluorescence (FL) region. Figure 1c demonstrates the effect of interaction between photons and phonons on the initial energy levels of ^7^F_1_, as it is observed in the spontaneous four-wave mixing (SFWM) region.

The photon Rabi frequency is defined as $G_{i}=-\mu_{H}H/\hbar$, where $\mu_{H}$ is the dipole moment between the |m〉 and |n〉 levels. Under a local magnetic field of the crystal, |m〉 and |n〉 are the CF splitting energy sub-levels of ^5^D_0_ and ^7^F_1_ in Figure 1(a2), and H is a magnetic field. The frequency detuning is $\Delta_{i}=\Omega_{mn}-\omega_{i}$; here, $\Omega_{mn}$ denotes the frequency of atomic transition between levels |m〉 and |n〉 and $\omega_{i}$ (i = 1,2) is the laser frequency. Relaxation of initial laser excitation with the assistance of crystal lattice phonons is mediated by the fine splitting of Eu^3+^ energy levels in the crystal field of BiPO_4_ (NaYF_4_). The phonon Rabi frequency is defined as $G_{p1}=-{\mu^{\prime }}_{H}H_{pi}/\hbar$, where $\mu_{H}$ is the dipole moment between levels |k〉 and |l〉 of the CF splitting energy level ^7^F_1_ in Figure 1(a1–a3). The phonon frequency detuning is $\Delta_{\mathrm{pi}}=\Omega_{kl}-\omega_{pi}$; here, $\Omega_{kl}$ denotes the resonant frequency between levels |k〉 and |l〉, $\omega_{pi}$ is the phonon field frequency, and $H_{pi}$ is the local magnetic field, which is determined from the vibration frequency of the crystal lattice state mode. Moreover, the phonon dressing term has been widely used in this article as it is linked with the phonons of different frequencies in the samples. This is because different lattice vibrations produce phonons of different frequencies coupled to different CF splitting levels [22,23,24].

**Figure 2:**
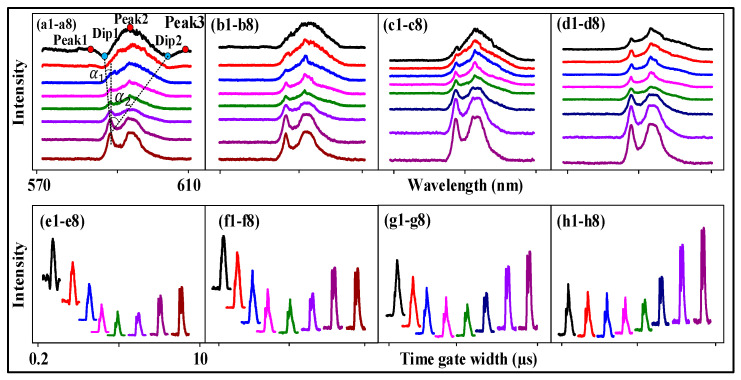
The excitation spectral intensity signals obtained for the (12:1) sample of Eu^3+^: BiPO_4_ by changing the time gate widths when *H*_1_ was scanned from 570 nm to 610 nm. Measured at PMT2 at high power and a temperature of 300 K. (**a**–**d**) Signals from the different time gate positions (700 ns, 900 ns, 1.1 µs and 1.6 µs) at time gate widths of 200 ns (**a1**–**d1**), 500 ns (**a2**–**d2**), 1 µs (**a3**–**d3**), 2 µs (**a4**–**d4**), 3 µs (**a5**–**d5**), 5 µs (**a6**–**d6**), 8 µs (**a7**–**d7**) and 10 µs (**a8**–**d8**). The connecting (**e**–**h**) correspond to (**a**–**d**), respectively.

Further, photomultiplier tubes 1 (PMT1) and 2 (PMT2) were placed at different angles/positions ($\theta_{1}$ and $\theta_{2}$), where $\theta_{1}<\theta_{2}$ (Figure 1d,e). PMT1 exhibits a strong SFWM response due to a large dressing Rabi frequency $G_{i}$, while PMT2 exhibits a strong FL response due to a large transverse de-phase rate $\Gamma_{ij}$. PMT3 exhibits a strong hybrid (FL+SFWM) response due to $\Gamma_{ij}\approx G_{i}$. The PMT angle $\theta_{i}$ (angle between the input laser beam and the output emission) is equal to the polarization angle $\theta_{pol}=\theta_{i}$ (Figure 2e). The polarization angle $\theta_{pol}$ is the angle between the laser polarization and output signal polarization. The photon dressing splitting angle $\theta_{q}$ is determined from the polarization angle (Figure 1d). Moreover, $\theta_{0i}$ (CF splitting angle) and $\theta_{pi}$ (phonon dressing splitting angle) are similar to $\theta_{i}$. The power, temperature, sample, time gate position and time gate width can control the polarization angle. This is because the spectral splitting of the z-axis in Figure 1d is in the same direction as the polarization of the laser field, phonon field and CF.

Figure 1d shows directional precession splitting (initial energy level splitting) induced by the dressing field and the crystal field. Figure 1e presents a schematic diagram of the experimental setup where PMTs are employed to detect the generated Stokes *(H*_S_), FL and hybrid signal, where $\theta_{i}$ is the angle between the PMTs and the sample, $\theta_{1}=60{}^{\circ}$, $\theta_{2}=45{}^{\circ}$ and $\theta_{3}=30{}^{\circ}$. The output Stokes (*H*_S_)/anti-Stokes (*H*_AS_) signals are generated under a phase-matched condition ($\kappa_{1}+{\kappa^{\prime }}_{1}=\kappa_{S}+\kappa_{AS}$) because of the interaction with the energy levels of Eu^3+^: BiPO_4_. PMT1 and PMT3 are precisely placed to detect a hybrid signal (FL + *H_s/_*_AS_) with dominant out-of-phase (different radiation direction) FL emissions, whereas PMT2 is placed near the sample to detect the generated in-phase (same radiation direction) spontaneous four-wave mixing (SFWM). The SFWM phase matching condition (K_1_ + K’_1_ = K_S_ + K_AS_) with angles ($\theta_{s}$, $\theta_{as}$, $\varphi_{s}$ and $\varphi_{as}$) is shown in Figure 1f. The $\theta_{s}$ and $\theta_{as}$ determine where Stokes and anti-Stokes are generated. When the $\theta_{s}$ and $\theta_{as}$ are small, the resulting stokes and anti-stokes are close to the sample location. The $\varphi_{s}$ and $\varphi_{as}$ determine the generation of Stokes and anti-Stokes amplitudes. When the $\varphi_{s}$ and $\varphi_{as}$ are small, the Stokes and anti-Stokes amplitude are large. There are two methods of achieving parity–time symmetry breaking (energy level splitting). The first is to change the size of the nanomaterial, and the second is to apply the external field, either constant [25] or alternating [26]. The effect of a constant electric field on the splitting of energy levels is known as a Stark effect, and the effect of a constant magnetic field is known as the Zeeman effect [27,28]. The CF induces energy level splitting (^7^F_1, MJ = ±1_,^7^F_1, MJ = 0_) through the static splitting alignment in the case of weak phonon dressing. Starting from the splitting in the crystal field, the coupling between photons and phonons further splits energy levels. Eventually, splitting of both fine structures was induced by the crystal field and induced by photon–phonon coupling.

The signal can be chosen from specific energy levels by changing the time gate position (time delay) and time gate width (integration duration). As a result, the time gate position may influence the FL/*H*s ratio. Since FL and *H*s decay at different rates, they can be recognized at the PMT using a boxcar time gate. The time gate width can also be used to change the number of photon emissions that are observed [29,30]. The observed signal originates from a single energy level with an unclear dressing effect if the time gate width is narrow enough.

### 2.1. Theoretical Model

The crystal field (*E*) splitting originates through a Stark field and demonstrates angular momentum precession around the *z*-axis (Figure 1(d1)). In general, angular momentum precession $\mu_{E}=-g\mu_{0}\;m_{J}$ causes splitting, which leads to the energy $\Delta E_{Z}=-g\mu_{0}m_{J}E\cos\theta_{0l}$. Here, $\Delta E_{Z}=m_{J}$, and $\Delta E_{Z}$ is a z-direction projection that represents discrete energy, which causes level splitting (*m_J_* = ±1, 0). In Figure 1(d3), g is a Landau factor for the crystal field, and $\mu_{0}$ is the electric moment. The CF splitting angle $\theta_{0l}$ reflects the quantum numbers (*m_J_*).

Dressing field (H) splitting or static splitting without flat alignment are shown in Figure 1(d2). Here, the intensity of the pure CF splitting level and the intensity of its dressing field CF level (*m_J_* = +1, 0, −1) become equal (J = 1). On the other hand, dressing field splitting produces angular precession $\mu_{E}=-g\mu_{o}\;m_{J}$ along the z-axis, as shown in Figure 1(d1). This leads to energy splitting $\Delta E_{Z}=-g\mu_{o}\;m_{J}E_{l}\cos\theta_{l}$ and $\Delta E_{Z}=-g\mu_{o}\;m_{J}E_{pl}\cos\theta_{pl}$, where $\theta_{l}$ and $\theta_{pl}$ correspond to quantum numbers $m_{J}$, $\mu_{o}$ is the electric moment and $\Delta E_{Z}$ is discrete energy, which causes the splitting of the dressing to assist CF levels in Figure 1(d2). Moreover, the photon dressing field further produces dressings with bright- and dark-state sublevels or hyperfine levels (Figure 1f). The internal splitting has two parts: one is the “density of state” (DOS), which is shown horizontally ($\Delta E_{x}=-g\mu_{E}m_{j}E\sin\theta_{0l}$) and represents the x-direction projection, and the other one is energy or frequency ($\Delta E_{Z}$), which is led by energy along the z-axis, as shown in Figure 1(d1). Here, DOS is inversely proportional to the frequency splitting gap.

**Figure 3:**
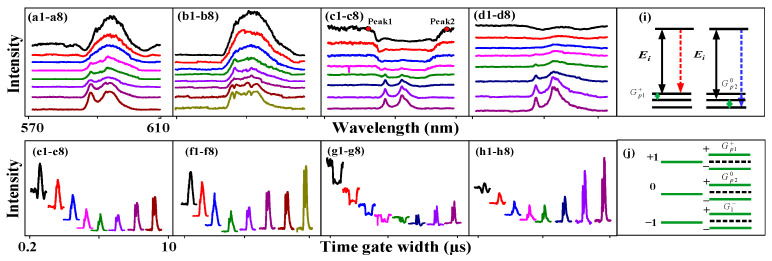
The excitation spectral intensity signals obtained for the (12:1) sample of Eu^3+^: BiPO_4_ by changing the time gate widths when *H*_1_ was scanned from 570 nm to 610 nm at high power and a temperature of 300 K: (**a**) Changing time gate widths (200 ns, 500 ns, 1 µs, 2 µs, 3 µs, 5 µs, 8 µs, 10 µs) measured at PMT2 and time gate position = 600 ns. (**b**) The PMT2 angle is larger than (**a**). (**c**) The time gate widths are changed and measured at PMT3 and time gate position = 1 µs. (**d**) The PMT3 angle is larger than (**c**). The connecting (**e**–**h**) correspond to (**a**–**d**). (**i**) Energy level splitting induced by different phonon dressings; (**j**) energy level splitting representing asymmetry, induced by photon dressing, phonon1 and phonon2 dressing.

**Figure 4:**
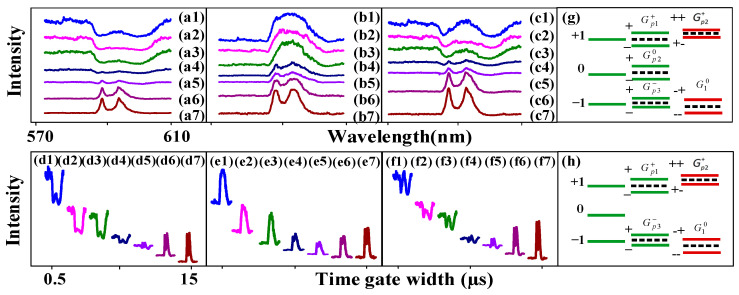
The excitation spectral intensity signals obtained for the near (12:1) sample of Eu^3+^: BiPO_4_ by changing the time gate widths when *H*_1_ was scanned from 570 nm to 610 nm at 100 ns time gate position, high power and a temperature of 300 K: (**a**) Changing time gate widths (500 ns, 1 µs, 2 µs, 3 µs, 5 µs, 10 µs, 15 µs) measured at PMT1. (**b**) Changing time gate widths measured at PMT2. (**c**) Changing time gate widths measured at PMT3. The connecting (**d**–**f**) Correspond to (**a**–**c**), respectively. (**g**,**h**) Energy levels representing asymmetry induced by different dressings.

**Figure 5:**
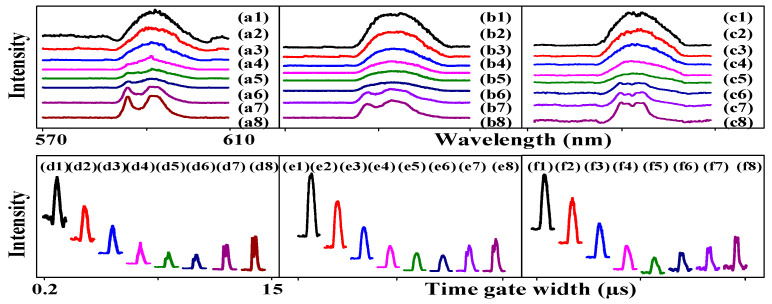
The excitation spectral intensity signals obtained for the different samples by changing the time gate widths (200 ns, 500 ns, 1 µs, 2 µs, 3 µs, 5 µs, 10 µs, 15 µs) when *H*_1_ was scanned at PMT2 from 570 nm to 610 nm at the 500 ns time gate position, high power and a 300 K temperature: (**a**) The broadband laser *H*_2_ is scanned (570 nm to 610 nm) for the (12:1) sample of Eu^3+^: BiPO_4_. (**b**) *H*_1_ is scanned (570 nm to 610 nm) for the (6:1) sample of Eu^3+^: BiPO_4_. (**c**) *H*_1_ is scanned (570 nm to 610 nm) for the (1:1/4) sample of Eu^3+^: NaYF_4_. (**d**–**f**) Connecting figures corresponding to (**a**–**c**), respectively.

**Figure 6:**
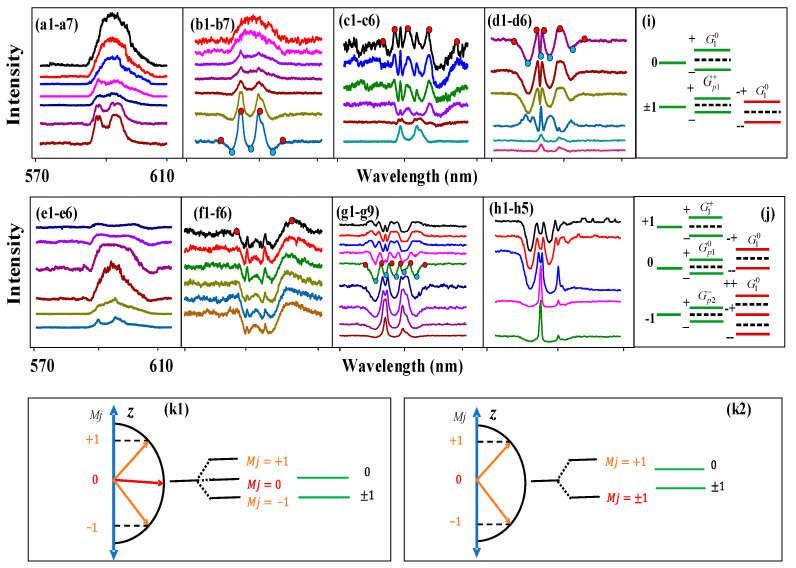
The excitation spectral intensity signals measured from the (12:1) sample of Eu^3+^: BiPO_4_ at PMT2: (**a**) The time gate widths are changed (200 ns, 500 ns, 1 µs, 2 µs, 5 µs, 10 µs, 15 µs) and *H*_1_ is scanned from 570 nm to 610 nm at the 500 ns time gate position, high power and a 300 K temperature. (**b**) *H*_2_ is scanned from 570 nm to 610 nm, and all other conditions are the same as in (**a**). (**c**) The time gate position is changed (500 µs, 580 µs, 640 µs, 1 ms, 3 ms, 5 ms) at the 3 µs time gate width, and all other conditions are the same as in (**a**). (**d**) The time gate positions are changed (20 µs, 50 µs, 100 µs, 500 µs, 1 ms, 5 ms) at a 200 ns time gate width, and all other conditions are the same as in (**b**). (**e**) The time gate positions are changed (50 ns, 100 ns, 200 ns, 500 ns, 1 µs, 2 µs) at the 200 ns time gate width, and all other conditions are the same as in (**a**). (**f**) The time gate width is changed (200 ns, 500 µs, 2 µs, 5 µs, 8 µs, 15 µs), and the laser is scanned from 570 nm to 610 nm at the 10 ms gate position and a 300 K temperature. (**g**) The power is changed from high to low, and *H*_2_ is scanned from 570 nm to 610 nm at a time gate width of 200 ns, time gate position of 500 µs and 300 K temperature. (**h**) The temperature is changed (280 k, 220 k, 150 k, 100 k, 77 k) and *H*_2_ is scanned from 570 nm to 610 nm at a time gate width of 200 ns, time gate position of 500 µs and high power. (**i**,**j**) Asymmetrical multi-level splitting for two dressings and three dressings, respectively. (**k1**,**k2**) Asymmetrical and symmetrical directional precession splitting.

The transition dipole moment between ^5^D_0_ and ^7^F_1_ can be calculated as $\mu_{kl}=\sum \langle {m^{\prime }}_{J}|m_{J}\rangle \mu_{o}$, where (${m^{\prime }}_{J}$ = 0) indicates excited-state splitting and ($m_{J}$ = −1, 0, +1) represents ground-state splitting. Similarly, the transition of phonons between sublevels can be calculated as $\mu_{p1}=\langle m_{J}=+1|m_{J}=0\rangle \mu_{o}$, $\mu_{p2}=\langle m_{J}=-1|m_{J}=0\rangle \mu_{o}$ and $\mu_{p3}=\langle m_{J}=+1|m_{J}=-1\rangle \mu_{o}$. The photon Rabi frequency $G_{i}≅|H_{i}||\mu_{kl}|\times \cos\theta_{l}/\hbar$ causes splitting of the magnetic dipole transition between ^5^D_0_ and ^7^F_1_ (Figure 1a). Meanwhile, the phonon Rabi frequency $G_{pl}≅|H_{pl}||\mu_{pl}|\times \cos\theta_{pl}/\hbar$ causes splitting among σ−, π and σ+ (Figure 1c). The external field changes the phase of angular momentum, which produces magnetic torque $\tau =\mu_{B}\times B$ and further results in an intensity splitting (Figure 2, Figure 3, Figure 4, Figure 5 and Figure 6).

By opening the *E*_1_ field, the $|\pm 1\rangle$ level of the dressed second-order FL via perturbation chain $\rho_{33}^{\left(0\right)}\overset{H_{1}}{\to }\rho_{32}^{\left(1\right)}\overset{H_{1}^{*}}{\to }\rho_{22}^{\left(2\right)}$ can be written as nested double dressing:

(1) $$\rho_{FL}^{-(2)}=\frac{|G_{1}^{-}(M){|}^{2}}{\left(\Gamma_{32}^{-}+i\Delta_{1}^{-})(\Gamma_{22}^{-}+|G_{1}^{-}(J){|}^{2}/(\Gamma_{32}^{-}+i\Delta_{1}^{-}+|G_{p2}^{0}(M){|}^{2}/(\Gamma_{31}^{0}+i\Delta_{1}^{-}-i\Delta_{p2}^{-})\right)}$$

where “−” represents the $|\pm 1\rangle$ level, and 0 represents the $|0\rangle$ level. H is the laser magnetic field and $\Gamma_{21}$ is the transverse decay rate between levels $|1\rangle$ and $|2\rangle$. The lifetime of FL is $\Gamma_{\mathrm{FL}}=\Gamma_{32}+\Gamma_{22}$. The dipole moment is $\mu_{Hi}=-g\mu J$ for the photon, and it is ${\mu^{\prime }}_{Hi}=-g\mu J^{\prime }$ for the phonon. Here, g is a Landau factor for the dressing field, μ is a constant and *i* = 1, 2, 3. The |0〉 level of the dressed second-order FL via perturbation chain $\rho_{11}^{\left(0\right)}\overset{H_{1}}{\to }\rho_{21}^{\left(1\right)}\overset{H_{1}^{*}}{\to }\rho_{22}^{\left(2\right)}$ can be written as nested double dressing:

(2) $$\rho_{FL}^{0(2)}=\frac{-|G_{1}^{0}(M){|}^{2}}{\left(\Gamma_{21}^{0}+i\Delta_{1}^{0})(\Gamma_{22}^{0}+|G_{1}^{0}{|}^{2}/(\Gamma_{21}^{0}+i\Delta_{1}^{0}+|G_{p2}^{0}{|}^{2}/(\Gamma_{31}^{0}+i\Delta_{1}^{0}+i\Delta_{p1}^{0}\right)}$$

By opening field *E*_1_, the $|\pm 1\rangle$ level of the dressed third-order density matrix element for *E_S_* ($\rho \prime_{S}^{\left(3\right)}$) and *E_AS_* ($\rho \prime_{AS}^{\left(3\right)}$) via perturbation chains $\rho_{33}^{\left(0\right)}\overset{H_{1}}{\to }\rho_{32}^{\left(1\right)}\overset{H_{AS}}{\to }\rho_{33}^{\left(2\right)}\overset{H_{1}^{*}}{\to }\rho_{32(S)}^{\left(3\right)}$ and $\rho_{33}^{\left(0\right)}\overset{H_{1}^{*}}{\to }\rho_{32}^{\left(1\right)}\overset{H_{S}}{\to }\rho_{33}^{\left(2\right)}\overset{H_{1}}{\to }\rho_{32(AS)}^{\left(3\right)}$, respectively, can be written as

(3) $$\rho_{S}^{-(3)}=\frac{-iG_{AS}^{-}G_{1}^{-}{G^{\prime }}_{1}^{-}}{\left(\Gamma_{32}^{-}+i\Delta_{1}^{-})(\Gamma_{33}^{-}+i\Delta_{1}^{-}-i\Delta_{AS}^{-}+|G_{1}^{0}{|}^{2}/(\Gamma_{32}^{0}+i\Delta_{1}^{-}-i\Delta_{AS}^{-}+i\Delta_{1}^{0}+|G_{p2}^{-}{|}^{2}/(\Gamma_{31}^{-}+i\Delta_{1}^{-}-i\Delta_{AS}^{-}+i\Delta_{p2}^{-}+i\Delta_{1}^{0}))(\Gamma_{32}^{-}+i\Delta_{1}^{-}-i\Delta_{AS}^{-}+i{\Delta^{\prime }}_{1}^{-}\right)}$$

(4) $$\rho_{AS}^{-(3)}=\frac{-iG_{S}^{-}G_{1}^{-}{G^{\prime }}_{1}^{-}}{\left(\Gamma_{32}^{-}+i{\Delta^{\prime }}_{1}^{-})(\Gamma_{33}^{-}+i{\Delta^{\prime }}_{1}^{-}-i\Delta_{S}^{-}+|G_{1}^{0}{|}^{2}/(\Gamma_{32}^{-}+i{\Delta^{\prime }}_{1}^{-}-i\Delta_{AS}^{-}+i\Delta_{1}^{0}+|G_{p2}^{-}{|}^{2}/(\Gamma_{31}^{-}+i{\Delta^{\prime }}_{1}^{-}-i\Delta_{S}^{-}+i\Delta_{p2}^{-}+i\Delta_{1}^{0}))(\Gamma_{32}^{-}+i\Delta_{1}^{-}-i\Delta_{S}^{-}+i{\Delta^{\prime }}_{1}^{-}\right)}$$

For the two dark states, the $|0\rangle$ level of the dressed third-order density matrix element for *E*_S_ ($\rho \prime_{S}^{\left(3\right)}$) and *E*_AS_ ($\rho \prime_{AS}^{\left(3\right)}$) via perturbation chains is $\rho_{11}^{\left(0\right)}\overset{H_{1}}{\to }\rho_{21}^{\left(1\right)}\overset{H_{AS}}{\to }\rho_{11}^{\left(2\right)}\overset{H_{1}^{*}}{\to }\rho_{21(S)}^{\left(3\right)}$ and $\rho_{11}^{\left(0\right)}\overset{H_{1}^{*}}{\to }\rho_{21}^{\left(1\right)}\overset{H_{S}}{\to }\rho_{11}^{\left(2\right)}\overset{H_{1}}{\to }\rho_{21(AS)}^{\left(3\right)}$, respectively, whose expressions can be written as follows:

(5) $$\rho_{S}^{0(3)}=\frac{-iG_{AS}^{0}G_{1}^{0}{G^{\prime }}_{1}^{0}}{\left(\Gamma_{21}^{0}+i\Delta_{1}^{0})(\Gamma_{11}^{0}+i\Delta_{1}^{0}-i\Delta_{AS}^{0}+|G_{10}^{0}{|}^{2}/(\Gamma_{21}^{0}+2i\Delta_{1}^{0}-i\Delta_{AS}^{0}+|G_{p1}^{+}{|}^{2}/(\Gamma_{10}^{+}+2i\Delta_{1}^{0}-i\Delta_{AS}^{0}+i\Delta_{p1}^{+}))(\Gamma_{21}^{0}+i\Delta_{1}^{0}+i{\Delta^{\prime }}_{1}^{0}-i\Delta_{AS}^{0}\right)}$$

(6) $$\rho_{AS}^{0(3)}=\frac{-iG_{S}^{0}G_{1}^{0}{G^{\prime }}_{1}^{0}}{\left(\Gamma_{21}^{0}+i{\Delta^{\prime }}_{1}^{0})(\Gamma_{11}^{0}+i{\Delta^{\prime }}_{1}^{0}-i\Delta_{S}^{0}+|G_{1}^{0}{|}^{2}/(\Gamma_{21}^{0}+i{\Delta^{\prime }}_{1}^{0}-i\Delta_{S}^{0}+i\Delta_{1}^{0}+|G_{p1}^{+}{|}^{2}/(\Gamma_{10}^{+}+i\Delta_{1}^{0}+i{\Delta^{\prime }}_{1}^{0}-i\Delta_{S}^{0}+i\Delta_{p1}^{+}))(\Gamma_{21}^{0}+i\Delta_{1}^{0}+i{\Delta^{\prime }}_{1}^{0}-i\Delta_{S}^{0}\right)}$$

When dressing splits the CF splitting levels into three levels (dressing energy level splitting), it is called dynamic splitting. The FL and SFWM of the form of the formula corresponding to the energy level are the same as in the static splitting, but the splitting mode is far different, which will not be described here.

By opening field *E*_1_, the dressed second-order FL via perturbation chain $\rho_{00}^{\left(0\right)}\overset{H_{1}}{\to }\rho_{20}^{\left(1\right)}\overset{H_{1}^{*}}{\to }\rho_{22}^{\left(2\right)}$ can be written as nested double dressing:

(7) $$\rho_{FL}^{+(2)}=\frac{|G_{1}^{+}(M){|}^{2}}{(\Gamma_{20}^{+}+i\Delta_{1}^{+}+|G_{p1}^{+}(J){|}^{2}/(\Gamma_{10}^{+}+i\Delta_{1}^{+}-i\Delta_{p1}^{+}+|G_{1}^{0}(M){|}^{2}/(\Gamma_{20}^{0}+i\Delta_{1}^{+}-i\Delta_{p1}^{+}+i\Delta_{1}^{0}))\Gamma_{22}^{+}}$$

For the two dark states of the dressed third-order density matrix element for *E_S_* ($\rho \prime_{S}^{\left(3\right)}$) and *E_AS_* ($\rho \prime_{AS}^{\left(3\right)}$) via perturbation chains $\rho_{00}^{\left(0\right)}\overset{H_{1}}{\to }\rho_{20}^{\left(1\right)}\overset{H_{AS}}{\to }\rho_{00}^{\left(2\right)}\overset{H_{1}^{*}}{\to }\rho_{20(S)}^{\left(3\right)}$ and $\rho_{00}^{\left(0\right)}\overset{H_{1}^{*}}{\to }\rho_{20}^{\left(1\right)}\overset{H_{S}}{\to }\rho_{00}^{\left(2\right)}\overset{H_{1}}{\to }\rho_{20(AS)}^{\left(3\right)}$, respectively, the expression can be written as

(8) $$\rho_{S}^{+(3)}=\frac{-iG_{AS}^{+}G_{1}^{+}{G^{\prime }}_{1}^{+}}{\left(\Gamma_{00}^{+}+i\Delta_{1}^{+}-i\Delta_{AS}^{+})(\Gamma_{20}^{+}+i\Delta_{1}^{+}+|G_{p1}^{+}{|}^{2}/(\Gamma_{10}^{+}+i\Delta_{1}^{+}-i\Delta_{p1}^{+}+|G_{1}^{0}{|}^{2}/(\Gamma_{20}^{0}+i\Delta_{1}^{+}-i\Delta_{p1}^{+}+i\Delta_{1}^{0}))(\Gamma_{20}^{+}+i\Delta_{1}^{+}+i{\Delta^{\prime }}_{1}^{+}-i\Delta_{AS}^{+}\right)}$$

(9) $$\rho_{AS}^{+(3)}=\frac{-iG_{S}^{+}G_{1}^{+}{G^{\prime }}_{1}^{+}}{\left(\Gamma_{00}^{+}+i{\Delta^{\prime }}_{1}^{+}-i\Delta_{S}^{+})(\Gamma_{20}^{+}+i{\Delta^{\prime }}_{1}^{+}+|G_{p1}^{+}{|}^{2}/(\Gamma_{10}^{+}+i{\Delta^{\prime }}_{1}^{+}-i\Delta_{p1}^{+}+|G_{1}^{0}{|}^{2}/(\Gamma_{20}^{0}+i{\Delta^{\prime }}_{1}^{+}-i\Delta_{p1}^{+}+i{\Delta^{\prime }}_{1}^{0}))(\Gamma_{20}^{+}+i{\Delta^{\prime }}_{1}^{+}+i\Delta_{1}^{+}-i\Delta_{S}^{+}\right)}$$

where + represents the $|+1\rangle$ level for the photon Rabi frequency and the phonon Rabi frequency, respectively. H is the laser magnetic field, and $\Gamma_{21}$ is the transverse decay rate between levels $|1\rangle$ and $|2\rangle$. The lifetime of FL is $\Gamma_{FL}=\Gamma_{21}+\Gamma_{22}$. The phonon dressings ${\left|G_{p2}\right|}^{2}$ and ${\left|G_{p3}\right|}^{2}$ can be added.

### 2.2. Experimental Results

We demonstrate the FL and SFWM asymmetry splitting, respectively, realized by changing the time gate position, time gate width, PMTs angle, PMTs position and sample. Here, we performed FL to SFWM evolution following the time domain decay curve at different time gate positions and time gate widths, which determine the value of the transverse de-phase rate Γ from large to small. Figure 2 shows the asymmetry evolution of the spectral FL signal obtained from (12:1) of Eu^3+^: BiPO_4_ at PMT2 and a 300 K temperature by changing the time gate width. The two dips in the FL signal near the time gate position (700 ns) are shown in Figure 2(a1). These can be explained using the two dressing terms $\left|G_{1}^{-}(M){|}^{2}/(\Gamma_{23}^{-}+i\Delta_{1}^{-}\right)$+$\left|G_{p1}^{+}(J){|}^{2}/(\Gamma_{10}^{+}+i\Delta_{1}^{+}-i\Delta_{p1}^{+}\right)$ from Equations (1) and (2). The ^7^F_1_ level is divided directly into m_j = −1_, m_j = 0_ and m_j = +1_ under the CF effect of the BiPO_4_ crystal and dressing (the dynamic splitting). Photon dressing leads to left dip1 (second-order splitting) in energy level $|-1\rangle$. Phonon dressing results in right dip2 (another form of splitting) in $|+1\rangle$. The left and right dips are symmetrical due to having the same dressing distribution. The intensity of dip1 and dip2 is 323 and 324, respectively. The dip intensity is basically equal due to FL asymmetry splitting. The slope of dip1 is S=dIm(ρ−)/dΔ1−=−Im((|G1−|2((|G1−|2i)/A2−i))/(Γ22−(A+|G1−|2/A)2)), where $A=\Gamma_{23}^{-}+i\Delta_{1}^{-}$. The slope of the $|-1\rangle$ level is $S_{-1}=-10.17$ in Figure 2(a1–a8). The slope of dip2 is S=dIm(ρ+)/dΔp1+=Re((|G1+|2|Gp1+|2)/(Γ22+(A+|Gp1+|2/B))2B2), where $A=\Gamma_{20}^{+}+i\Delta_{1}^{+}$ and $B=\Gamma_{10}^{+}+i\Delta_{1}^{+}-i\Delta_{p1}^{+}$. The slope of the $|+1\rangle$ level is 1.02. The distance between peak1 and peak2 is 10 nm. The distance between peak2 and peak3 is 14.5 nm.

Moreover, as the time gate width increases from Figure 2(a1–a3), the two dips decrease. The dressing dips decrease due to the increase in $\Delta_{p1}^{+}$ and $\Delta_{1}^{-}$. When the time gate width increases from 1 µs to 10 µs nm in Figure 2(a3–a8), respectively, the linewidth of the spectrum signal continuously decreases from 32 nm to 15 nm due to the strong CF splitting (the static splitting). Therefore, the FL asymmetry splitting can be controlled by the time gate width. Theoretically, the maximum of three emission peaks can be observed in Eu^3+^: BiPO_4_ due to three fine-structure energy levels. However, in our experiment, we observed only two peaks shown in Figure 2(a6), suggesting that ^7^F_1_, _MJ = ±1_ and ^7^F_1_, _MJ = 0_ are indistinguishable and distinguishable, respectively.

Further, a broad peak is shown in Figure 2(b1) due to the dominance of CF splitting. The two dressing dips disappeared from Figure 2(a1–b1) due to the increase in the time gate position reducing $\Gamma_{10}^{+}$. The fluorescence asymmetry ratio is defined as $R_{FL}=S_{CF}/S_{D}$, where the $S_{CF}$ is the area of the peak from crystal field splitting. The $S_{D}$ is the area of the dip/peak from the photon–phonon dressing. The FL asymmetry ratio is $c_{\mathrm{FL}}=(18.16-13.99)/18.16=22.96\%$ from Figure 2(b1–b8). The slope from the $|-1\rangle$ level is S=dIm(ρ−)/dΔ1−=−12.12. The slope from the $|+1\rangle$ level is S+1=dIm(ρ+)/dΔ1+=1.063 in Figure 2(b1–b8). Therefore, the FL asymmetry splitting can be changed by changing the time gate position. The competitive relationship between photon dressing and CF splitting is shown in Figure 2c,d. The right peak AT splitting is seen in Figure 2(c8) due to the dominance of the CF splitting at a larger time gate width. The FL asymmetry ratio is 9.26% in Figure 2c, and the slope of the $|-1\rangle$ and $|+1\rangle$ levels is S=dIm(ρ−)/dΔ1−=−34.26. The symmetry is shown in Figure 2d, and thus, the slope on both sides is near infinity due to strong CF splitting.

Additionally, the connecting Figure 2e corresponds to the overlapping Figure 2a. When the time gate width is increased, the background signal decreases and then balances. This is because the disorder signal is dominant at a narrow time gate width (Figure 2(e1–e5)). The frequency domain signal is averaged based on the boxcar time gate position. The more disordered the non-resonant signal, the more serious the mutual offset (out of phase), resulting in a background signal (Figure 2(e1–e5)). The FL signal and the SFWM signal reach a state of competitive equilibrium (hybrid) in Figure 2(e5–e8), so the background signal does not change. Figure 2f–h shows a similar result to Figure 2e.

Next, we discuss FL and SFWM asymmetry splitting at the different PMT positions and angles. Figure 3 shows the spectral signal of the FL obtained from the (12:1) sample of Eu^3+^: BiPO_4_, which was collected by PMT2 and PMT3 by changing the time gate width at a temperature of 300 K. We know that placing PMT1 at a small angle results in large splitting ($\cos\theta_{l}$ in Figure 1d–f), and placing PMT2 at a large angle results in small splitting ($\cos\theta_{1}>\cos\theta_{2}$, $G_{1}≅|H_{1}||\mu_{kl}|\times \cos\theta_{1}/\hbar >G_{2}≅|H_{2}||\mu_{kl}|\times \cos\theta_{2}/\hbar$). The distance between peak1 and peak2 is 10.76 nm in Figure 3a. The distance between peak2 and peak3 is 15.41 nm. The intensities of dip1 and dip2 are 495.75 and 496.61, respectively. The FL asymmetry ratio is 42.3%. The slopes of the $|-1\rangle$ and $|+1\rangle$ levels are −8.11 and 0.95, respectively. The PMT angle is reduced in Figure 3b compared with Figure 3a. The FL signal is not controlled by the PMT angle in Figure 3(b1–b4). However, the more in-phase SFWM signals are collected at smaller angles. The dressing is not shown in the FL signal, while AT splitting is seen in the SFWM signal. This can be explained by nested double dressing $\left|G_{1}^{-}{|}^{2}/(\Gamma_{23}^{-}+i\Delta_{1}^{-}-i\Delta_{AS}^{-}\right)$+$\left|G_{p1}^{0}{|}^{2}/(\Gamma_{13}^{0}+i\Delta_{1}^{0}-i\Delta_{p1}^{0}-i\Delta_{AS}^{0}\right)$ from Equations (3) and (4). In Figure 3(b7), AT splitting is increased and decreased in the left and right peaks, respectively, when the time gate width increases. This shows that circular polarization and linear polarization have different effects on AT splitting. The FL asymmetry ratio is 28.18%. The slopes of the $|-1\rangle$ and $|+1\rangle$ levels are −9.248 and 0.994, respectively. These results suggest the presence of a routing phenomenon, and our proposed asymmetry spectral router model based on the dressing Rabi frequency splitting presented in Figure 1g. The experiment setup presented in Figure 1e is used to realize the asymmetry spectral router (Figure 1g), where the Eu^3+^: BiPO_4_ crystal behaves as a router with the *E*_1_ beam as ist input (a_in_); *E*_2_ is a control signal, and a_oft he_the outpft hethe router detected at PMTs. Here, we used the channel equalization ratio ($P=1-\sqrt{\sum_{1}^{1-N}{\left(b_{i}-a\right)}^{2}/a}$) to measure the de-multiplexing, where N corresponds to the number of peaks after splitting, a refers to the area of one peak after splitting, and $b_{i}$ represents the splitting distance between the adjacent peaks, respectively. The channel equalization ratio for Figure 3b was calculated as *P* = 96.6%, which is a lot higher than the results proposed for other atomic-like media.

Moreover, Figure 3c,d show a spectral signal at the 1 µs time gate position and near PMT3. We can show FL (Figure 3(c4)) and SFWM (Figure 3(c1)) asymmetry (same DOS) through evolution. The dressing dip is seen in Figure 3(c1), which can be explained by the three nested dressings $\left|G_{1}^{-}(M){|}^{2}/(\Gamma_{23}^{-}+i\Delta_{1}^{-}\right)$+$\left|G_{p2}^{0}(M){|}^{2}/(\Gamma_{31}^{0}+i\Delta_{1}^{0}+i\Delta_{p2}^{0}\right)$+$\left|G_{p1}^{+}(M){|}^{2}/(\Gamma_{10}^{+}+i\Delta_{1}^{+}-i\Delta_{p1}^{+}\right)$ in Equations (5) and (6). The coupling of the phonon1 dressing with the $|-1\rangle$ level leads to the left dip in Figure 3(c1) and the coupling of the phonon2 dressing with the $|+1\rangle$ level leads to the right dip. The coupling of the photon dressing with the $|0\rangle$ level leads to the middle dip (Figure 3i). The left and right dips are basically symmetrical, so the corresponding dressing distribution is the same. The dip depth ranged from 404 (Figure 3(c1)) to 110 (Figure 3(c4)); this is because $\Delta_{p1}^{+}$ and $\Delta_{p2}^{0}$ become small, and thus, the phonon dressing becomes small. The FL asymmetry ratio is 49.4% in Figure 3c. The slopes of the $|-1\rangle$ and $|+1\rangle$ levels are −5.9 and 0.935, respectively. Similar to Figure 3c, the FL asymmetry ratio is 11.99% in Figure 3d due to photon–phonon dressing from Equation (7). The slopes of the $|-1\rangle$ and $|+1\rangle$ levels are 24.2 and 1.156, respectively. The FL asymmetry ratio in Figure 3d is 13% due to the coupling of the one-photon dressing ($|G_{1}^{-}{|}^{2}e^{i\Delta \varphi_{1}}$) with the $|-1\rangle$ level (Figure 3j). The slopes of $|-1\rangle$ and $|+1\rangle$ are −24.16 and 66.66, respectively. Therefore, the strong FL asymmetry splitting can be controlled by the small PMT angle due to the greater photon–phonon dressing. The SFWM asymmetry ratio is defined as $R_{SF}=S_{CF}/S_{M}$, where the $S_{CF}$ is the area of the peak from crystal field splitting. The $S_{M}$ is the area of the multi-dips from the photon–phonon dressing. The $R_{SF}$ is about 32.2% from Figure 3(c1–c8). Therefore, the SFWM is more sensitive to phonon dressing.

Further, the connecting Figure 3e–h correspond to the overlapping Figure 3a–d. It is also shown that the SWFM signal is dominant, and the background signal is stronger. The ratios of the maximum signal and the FL are 2515/672 (Figure 3(e1–e5)), 2651/644 (Figure 3(f1–f4)), 2651/103 (Figure 3(g1–g5)) and 367/195 (Figure 3(h1–h5)). The ratios of the maximum signal and the SWFM are 1808/672 (Figure 3(e5–e8)), 644/460 (Figure 3(f4–f8)), 456/103 (Figure 3(g5–g8)) and 1813/367 (Figure 3(h5–h8)).

Next, Figure 4 shows different FL/SFWM asymmetries splitting at different PMT positions. Under the same conditions, the dressing has a great impact on the FL signal (peak or dip) collected by changing the location of the PMT. The alignment of the dips in Figure 4(a1) can be explained by the five photon–phonon1–phonon2 dressings $\left|G_{p3}^{-}{|}^{2}/(\Gamma_{03}^{-}+i\Delta_{1}^{-}-i\Delta_{p3}^{-}+|G_{1}^{0}{|}^{2}/(\Gamma_{23}^{0}+i\Delta_{1}^{-}-i\Delta_{p3}^{-}+i\Delta_{1}^{0}\right)$ from Equations (3) and (4), $\left|G_{p2}^{0}(M){|}^{2}/(\Gamma_{31}^{0}+i\Delta_{1}^{0}+i\Delta_{p2}^{0}\right)$ from Equations (5) and (6) and $\left|G_{p1}^{+}{|}^{2}/(\Gamma_{10}^{+}+i\Delta_{1}^{+}-i\Delta_{p1}^{+}+|G_{p2}^{0}{|}^{2}/(\Gamma_{13}^{0}+i\Delta_{1}^{+}-i\Delta_{p1}^{+}-i\Delta_{p2}^{0}\right)$ from Equations (8) and (9) due to the PMT being located at a shorter distance (big $\varphi_{1}$, $1/\cos\varphi_{1}$). When the depth of the dip is the same, if the dip linewidth is smoother, then the dressing is larger. Although the dip is flatter in Figure 4(a1,a2), the depth of the dips decreases, so the dressing becomes weak. The distance between peak1 and peak2 is 26.3 nm. The FL asymmetry ratio is 48.86% in Figure 4a. The slopes of the $|-1\rangle$ and $|+1\rangle$ levels are −17.54 and 1.11, respectively.

Further, a broad peak is seen in Figure 4(b1) due to the dominance of CF splitting (small $\varphi_{2}$, $1/\cos\varphi_{2}$). As the time gate width increases to 15 µs, the photon dressing becomes weak, and the CF splitting becomes strong. The FL asymmetry ratio is 26.38% in Figure 4b. The slopes of the $|-1\rangle$ and $|+1\rangle$ levels (Figure 4g) are −19.49 and 1.13, respectively. Similar to Figure 3(a1), the two dips in Figure 4(a1) are deep because there is less $\Delta_{p1}^{+}$. The distance between peak1 and peak2 is 9.97 nm. The distance between peak2 and peak3 is 15.77 nm. The two Fano dips resulting from the middle $\varphi_{3}$ ($1/\cos\varphi_{3}$) are shown in Figure 4(c1). The FL asymmetry ratio is 45.82% in Figure 4c. Therefore, the FL asymmetry splitting is strong at the near-PMT position due to the dominance of photon–phonon dressing. The slope of the $|-1\rangle$ and $|+1\rangle$ levels (Figure 4h) are −10.67 and 1.185, respectively. The connecting Figure 4d–f correspond to the overlapping Figure 4a–c. The ratios of the maximum and minimum FL signal are 1240/202 (Figure 4(d1–d5)), 1782/608 (Figure 4(e1–e5)) and 994/277 (Figure 4(f1–f5)). The ratios of the maximum and minimum SFWM signals are 1087/202 (Figure 4(d5–d7)), 1566/608 (Figure 4(e5–e7)) and 1683/277 (Figure 4(f5–f7)).

Next, we discuss the different FL asymmetries splitting at different bandwidths and samples. The evolution of the FL signal obtained from different samples collected with the PMT2 and with changing time gate widths at a temperature of 300 K is shown in Figure 5a. The time gate position is fixed at 500 ns. Similar to Figure 2(a1), the distance between peak1 and peak2 is 10.76 nm. The distance between peak2 and peak3 is 15.41 nm. The FL asymmetry ratio is 43.98% in Figure 5(a1) due to the presence of more phonon dressing (small $\Delta_{pi}^{12:1}$, $1/\Delta_{pi}^{12:1}$). The slopes of the $|-1\rangle$ and $|+1\rangle$ levels are −6.78 and 0.94, respectively. The FL asymmetry ratio is 31.69% in Figure 5(b1). We obtained a stronger FL asymmetry splitting for (12:1) than for (6:1) Eu^3+^: BiPO_4_ due to the higher phonon frequency and stronger dressing. The slopes of the $|-1\rangle$ and $|+1\rangle$ levels are −7.547 and 0.96, respectively. Unlike the Eu^3+^: BiPO_4_ sample in Figure 5(b1), whose energy level cannot be cleaved, the CF splitting can be cleaved for the Eu^3+^: NaYF_4_ sample in Figure 5(c1). The distance between peak1 and peak2 is 10.09 nm. The distance between peak2 and peak3 is 15.28 nm. The FL asymmetry ratio is 50.2%. The slopes of the $|-1\rangle$ and $|+1\rangle$ levels are −2.54 and 1.23, respectively. Therefore, the FL asymmetry splitting is the strongest for Eu^3+^: NaYF_4_ because its dressing is the strongest.

Moreover, through a comparison of the three samples, we can see that the FL signal shows strong dressing in Figure 5a. The CF is well split. The intensity of the phonons in the sample in Figure 5b is greater than in Figure 5a, but the dressing is weak due to the middle $\Delta_{pi}^{6:1}$($1/\Delta_{pi}^{6:1}$). Different samples are compared under the same conditions, and it can be seen that the dressing is stronger for the 12:1 sample in Figure 5a, and the CF splitting, the dip depth and the alignment are better. The photon dressing for the 6:1 sample is shown in Figure 5b due to the dressing damage. The dressing dips change significantly in Figure 5a,b due to the action of phonons. Meanwhile, the dressing becomes weak in Figure 5c (large $\Delta_{pi}^{1:1/4}$, $1/\Delta_{pi}^{12:1}>1/\Delta_{pi}^{6:1}>\Delta_{pi}^{1:1/4}$). The energy level splitting is caused by asymmetry for the Eu^3+^: NaYF_4_ sample. Therefore, the change in the dressing dip is not obvious from Figure 5a–c due to the asymmetry of the crystal. This indicates that the phonon plays a more important role than the crystal asymmetry, which is reflected in asymmetry, division, strength and back bottom. The connecting Figure 5d–f corresponds to the overlapping Figure 5a–c. The ratios of the maximum signal and the FL are 2783/857 (Figure 5(d1–d6)), 3341/820 (Figure 5(e1–e6)) and 2480/672 (Figure 5(f1–f6)). The ratios of the maximum signal and the SWFM are 1808/857 (Figure 5(d6–d8)), 1745/820 (Figure 5(e6–e8)) and 1379/672 (Figure 5(f6–f8)).

Next, we discuss the differences in SFWM asymmetry splitting under the different conditions. Figure 6 shows the signals generated from the Eu^3+^: BiPO_4_ using different experimental parameters in PMT2. The broad peak in Figure 6a is due to the narrowband excitation, leading to weak photon–phonon dressing $\left|G_{p1}^{+}(J){|}^{2}/(\Gamma_{10}^{+}+i\Delta_{1}^{+}-i\Delta_{p1}^{+}+|G_{1}^{0}(M){|}^{2}/(\Gamma_{20}^{0}+i\Delta_{1}^{+}-i\Delta_{p1}^{+}+i\Delta_{1}^{0}\right)$ in Equation (7). A broad peak is still shown in Figure 6(b1–b5) at the small time-gate width because the broadband excitation couples more high-frequency phonons. However, two dressing dips can be seen in Figure 6(b6,b7) resulting from two dressings: $\left|G_{1}^{-}{|}^{2}/(\Gamma_{23}^{-}+i\Delta_{1}^{-}-i\Delta_{AS}^{-}\right)$+$\left|G_{1}^{0}{|}^{2}/(\Gamma_{21}^{0}+i\Delta_{1}^{0}-i\Delta_{AS}^{0}\right)$ from Equations (3)–(6). When the dressing increases, the linewidth also increases. Because the $\Delta_{1}^{-}$ decreases and the CF splitting is dominant, we can see two peaks on the left and right in Figure 6(b7). The left and right peaks are almost completely symmetrical due to the weak dressing and the strong CF splitting. The photon dressing ($|G_{1}^{-}{|}^{2}$ and $|G_{1}^{0}{|}^{2}$) leads to second-order splitting at the energy levels $|\pm 1\rangle$ and $|0\rangle$ (Figure 6i), respectively. The conditions of first-order splitting enhancement are $a_{1}=(2\Delta_{1}^{-}+\sqrt{{\left(2\Delta_{1}^{-}\right)}^{2}+4(\Gamma_{23}^{-}\Gamma_{23}^{-}-{\left(\Delta_{1}^{-}\right)}^{2}+{|G}_{1}^{-}{|}^{2})})/2$ and $a_{2}=(2\Delta_{1}^{-}-\sqrt{{\left(2\Delta_{1}^{-}\right)}^{2}+4(\Gamma_{23}^{-}\Gamma_{23}^{-}-{\left(\Delta_{1}^{-}\right)}^{2}+{|G}_{1}^{-}{|}^{2})})/2$. The distance between peak1 and peak2 is 5.43 nm. The distance between peak3 and peak4 is 8.06 nm. The distance between peak2 and peak3 is 5.97 nm. The FL asymmetry ratio is 31.83% in Figure 6a. The slopes of the $|-1\rangle$ and $|+1\rangle$ levels are −4.35 and 0.86, respectively. The FL asymmetry ratio is 33.22% in Figure 6b; thus, the slopes of the two levels are near infinity. Figure 6a corresponds to Figure 6(k1), and Figure 6b corresponds to Figure 6(k2). When the $|\pm 1\rangle$ energy level is divided into $|+1\rangle$ and $|-1\rangle$ (Figure 6j) through weak phonon dressing, the $|0\rangle$ becomes the center of the energy levels. As a result, a strong asymmetry peak can be seen in Figure 6a. Therefore, the FL asymmetry splitting is strong under broadband excitation due to the presence of more phonon dressing.

Figure 6c shows the luminescence spectra of SFWM obtained at the far time gate position. The multi-Fano dip (Figure 6c) can be explained by the five-nested dressing $|G_{p2}^{-}{|}^{2}+|G_{1}^{0}{|}^{2}+|G_{p1}^{0}{|}^{2}/|G_{1}^{0}{|}^{2}+|G_{1}^{+}{|}^{2}$. Because the SFWM signal is sensitive to phonon dressing, the SFWM asymmetry ratio is 52.17% in Figure 6c. Under the same conditions, three multi-dips can be seen in Figure 6d as a result of three SFWM phonon dressings ($|G_{1}^{0}{|}^{2}/(\Gamma_{20}^{0}+i\Delta_{1}^{0})+|G_{pi}^{0}{|}^{2}$) when the time gate position is changed. The left and right peaks are basically symmetrical in Figure 6(d1). The conditions of first-order splitting enhancement are $a_{1}=(2\Delta_{p1}^{-}+\sqrt{{\left(2\Delta_{p1}^{-}\right)}^{2}+4(\Gamma_{23}^{-}\Gamma_{13}^{-}-{\left(\Delta_{p1}^{-}\right)}^{2}+{|G}_{p1}^{-}{|}^{2})})/2$ and $a_{2}=(2\Delta_{p1}^{-}-\sqrt{{\left(2\Delta_{p1}^{-}\right)}^{2}+4(\Gamma_{23}^{-}\Gamma_{13}^{-}-{\left(\Delta_{p1}^{-}\right)}^{2}+{|G}_{p1}^{-}{|}^{2})})/2$ for the real part, and $b_{1}=-(\Gamma_{23}^{-}+\Gamma_{13}^{-})+\sqrt{{\left(\Gamma_{23}^{-}+\Gamma_{13}^{-}\right)}^{2}-4(\Gamma_{23}^{-}\Gamma_{13}^{-}-{\left(\Delta_{p1}^{-}\right)}^{2}+{|G}_{p1}^{-}{|}^{2})}/2$ and $b_{2}=-(\Gamma_{23}^{-}+\Gamma_{13}^{-})-\sqrt{{\left(\Gamma_{23}^{-}+\Gamma_{13}^{-}\right)}^{2}-4(\Gamma_{23}^{-}\Gamma_{13}^{-}-{\left(\Delta_{p1}^{-}\right)}^{2}+{|G}_{p1}^{-}{|}^{2})}/2$ for the imaginary part. The conditions of second-order splitting enhancement are $c_{1}=(2a_{1}+\Delta_{p1}^{-}+\Delta_{1}^{0})+\sqrt{{\left(2a_{1}+\Delta_{p1}^{-}+\Delta_{1}^{0}\right)}^{2}-4(b_{1}\Gamma_{23}^{0}+a_{1}^{2}+\Delta_{p1}^{-}+\Delta_{1}^{0})}/2$ and $c_{2}=(2a_{2}+\Delta_{p1}^{-}+\Delta_{1}^{0})-\sqrt{{\left(2a_{2}+\Delta_{p1}^{-}+\Delta_{1}^{0}\right)}^{2}-4(b_{2}\Gamma_{23}^{0}+a_{1}^{2}+\Delta_{p1}^{-}+\Delta_{1}^{0})}/2$. The conditions of suppression are $\lambda_{\pm +}=|1+\rangle$. The conditions of dip1 are $\lambda_{-}^{+}=(\Delta_{p1}^{-}-\sqrt{{\left(\Delta_{p1}^{-}\right)}^{2}+4{\left|G_{p1}^{-}\right|}^{2}})/2$. The conditions of dip2 are $\lambda_{+}^{+}=(\Delta_{1}^{-}+\sqrt{{\left(\Delta_{1}^{-}\right)}^{2}+4{\left|G_{1}^{-}\right|}^{2}})/2$. The distance between peak1 and peak3 is 8.53 nm. The conditions of first-order splitting enhancement are $a_{3}=(2\Delta_{1}^{0}+\sqrt{{\left(2\Delta_{1}^{0}\right)}^{2}+4(2\Gamma_{20}^{0}-{\left(\Delta_{1}^{0}\right)}^{2}+{|G}_{1}^{0}{|}^{2})})/2$ and $a_{4}=(2\Delta_{1}^{0}-\sqrt{{\left(2\Delta_{1}^{0}\right)}^{2}+4(2\Gamma_{20}^{0}-{\left(\Delta_{1}^{0}\right)}^{2}+{|G}_{1}^{0}{|}^{2})})/2$ at the $|0\rangle$ level. The position of the $|0\rangle$ level (dip4) satisfies the suppression conditions $\lambda_{\pm }=|0\rangle$. The distance between peak4 and peak5 is 5.78 nm; the distance between peak3 and peak4 is 5.44 nm. The intensity of dip1 is 2166.53 nm, and that of dip2 is 1666.1 nm. The slopes of dip1 and dip4 are −4.6 and 4.97, respectively. The SFWM asymmetry ratio is 53.21% in Figure 6d due to strong phonon dressing resulting from broadband excitation.

The energy levels splitting are shown in Figure 6(k1,k2), corresponding to Figure 6c,d, respectively. Figure 6e shows the FL signal. The FL asymmetry ratio is 10.05%. The slopes of the $|-1\rangle$ and $|+1\rangle$ levels are −5.77 and 1.09, respectively. Compared to Figure 6a, asymmetry is more sensitive to the time gate position in Figure 6e. Compared with Figure 6c, the SFWM symmetry ratio is 0.2% in Figure 6f. It can be seen that the SFWM asymmetry is more sensitive to the time gate position due to the phonon dressing reaching the maximum. Compared with Figure 6c, the number of dressings is the same, but the dressing is larger (Figure 6(k1)), resulting in the multi-Fano dips in Figure 6f. It can be confirmed again that when the time gate position is near, the asymmetry is controlled by the time gate width. However, at a large time gate width, the asymmetry is controlled by the time gate position. The left and right dressing dips are asymmetry, which is related to the circular polarization. Similarly, the distance between peak1 and peak2 is 16.59 nm, and the asymmetry is only small, as shown in Figure 6f.

Figure 6g shows the luminescence spectra when the power is changed from high to low. Compared to Figure 6(g9) at low power, Figure 6(g1) shows multi-Fano dips at high power due to strong in-phase constructive dressing in the SFWM region. The distance between peak1 and peak3 is 8.19 nm. The distance between peak4 and peak6 is 11.22 nm. The middle energy levels of |0> and |±1> come from dressing-assistance crystal splitting (Figure 6(k2)). The distance between peak3 and peak4 is 3.28 nm. Therefore, the slopes of dip1 and dip5 are −4.44 and 4.43, respectively. The two slopes show opposite numbers. Figure 6h shows the spectral signal resulting from the change in temperature. Compared to the low temperature (smaller $G_{pl}$ and larger $\Gamma_{ij}$) in Figure 6(h5), the high temperature in Figure 6(h5) shows strong multi-Fano dips due to in-phase constructive dressing. The distance between peak1 and peak3 is 8.1 nm. The distance between peak3 and peak4 is 3.94 nm. The distance between peak4 and peak5 is 2.48 nm. Therefore, the slopes of dip1 and dip5 are −4.51 and 4.78, respectively. The two slopes show opposite numbers. By comparing Figure 6g,h, we can conclude that, upon changing the power and temperature, the center of the signal is basically unchanged and has no asymmetry in Figure 6h. The amplitude and Rabi frequency are controlled by the power and temperature. The detuning is controlled by changing the time gate position and time gate width. Therefore, the FL and SFWM asymmetry is not changed.

## 3. Conclusions

This study proved that the spectral asymmetry of out-of-phase fluorescence (FL) and in-phase spontaneous four-wave mixing (SFWM) ion-doped microcrystals is controlled through the competition between single-photon and double photon–phonon dressing and CF splitting, which can be adjusted by tuning the parameters of the time gate position, time gate width, PMT angle, PMT position, sample and bandwidth. Moreover, the FL and SFWM symmetry remains unchanged when the temperature and power are controlled. The asymmetry includes out-of-phase FL and in-phase SFWM. When the time gate width is increased, the asymmetry with the FL signal becomes acute. As the time gate position increases, the asymmetry with the SFWM signal is significantly enhanced. The size of the ratio between the dressing Rabi frequency and de-phase rate can regulate the strength of asymmetry splitting. Moreover, the experiment results suggest that the FL asymmetry splitting maximum ratio reaches 50.2%. However, the SFWM asymmetry splitting minimum ratio reaches 52.17%. Therefore, compared with out-of-phase FL, in-phase SFWM is more sensitive to phonon dressing. Further, the asymmetry between the effects of photon–phonon dressing and CF on the fine structure of FL and SFWM can be used for an optical router with a channel equalization ratio of ≈96.6%.

## Data Availability

The data underlying the results presented in this paper are not publicly available at this time but may be obtained from the authors upon reasonable request.
